# Food Composition Databases: Does It Matter to Human Health?

**DOI:** 10.3390/nu13082816

**Published:** 2021-08-17

**Authors:** Amélia Delgado, Manel Issaoui, Margarida C. Vieira, Isabel Saraiva de Carvalho, Anthony Fardet

**Affiliations:** 1 Mediterranean Institute for Agriculture, Environment and Development, University of Algarve, 8005-139 Faro, Portugal; mvieira@ualg.pt (M.C.V.); icarva@ualg.pt (I.S.d.C.); 2 Department of Biotechnology, Faculty of Science and Technology of Sidi Bouzid, University of Kairouan, Sidi Bouzid 9100, Tunisia; manelissaoui80@gmail.com; 3 Department of Food Engineering, Superior Institute of Engineering, University of Algarve, 8005-139 Faro, Portugal; 4 FSLab—Food Science Laboratory, Faculty of Sciences and Technology, University of Algarve, 8005-139 Faro, Portugal; 5 Unité de Nutrition Humaine, INRAE, Route de Theix, 63122 Saint-Genès-Champanelle, France; anthony.fardet@inrae.fr

**Keywords:** food data, natural substances, health promotion, sustainable foods, national food composition databases, one health

## Abstract

Food provides humans with more than just energy and nutrients, addressing both vital needs and pleasure. Food habits are determined by a wide range of factors, from sensorial stimuli to beliefs and, once commanded by local and seasonal availability, are nowadays driven by marketing campaigns promoting unhealthy and non-sustainable foodstuffs. Top-down and bottom-up changes are transforming food systems, driven by policies on SDGs and by consumer’s concerns about environmental and health impacts. Food quality, in terms of taste, safety, and nutritional value, is determined by its composition, described in food composition databases (FDBs). FDBs are then useful resources to agronomists, food and mechanical engineers, nutritionists, marketers, and others in their efforts to address at maximum human nutrient needs. In this work, we analyse some relevant food composition databases (viz., purpose, type of data, ease of access, regularity of updates), inspecting information on the health and environmental nexus, such as food origin, production mode as well as nutritional quality. The usefulness and limitations of food databases are discussed regarding what concerns sustainable diets, the food ‘matrix effect’, missing compounds, safe processing, and in guiding innovation in foods, as well as in shaping consumers’ perceptions and food choices.

## 1. Introduction

Food databases (FDB), or more correctly food composition databases, contain detailed information on the nutritional composition of foods and on other relevant compounds (e.g., polyphenols, phytic acid). Food components primarily determine nutritional features and, in some cases, quality aspects. For example, polyphenols, which are abundant in plants, are often associated to bitter taste and astringency sensation of foods [1], while acting in favour of food safety by inhibiting foodborne pathogens and spoilage microbes. Polyphenols can be intentionally added to foods for their bioactive properties [2,3,4] or they can be key natural components, as happens in table olive fermentation [5,6]. During the spontaneous fermentation process, olive’s polyphenols help to select the suitable microbial populations, resulting in taster and safer foodstuffs.

The applications of FDBs have been greatly evolving and, consequently, the awareness on some of their limitations. Firstly, FDBs consisted of printed tables listing the nutritional composition of selected foods, usually from a certain country and only available to a few specialists. Today, the most popular FDBs are open access online comprehensive datasets and resources, which may provide answers to simple queries or the download of large datasets; for this reason, the main FDBs are compatible among them and with many interface applications. Up to date food composition data are of capital importance for estimations in relation to nutrition and public health, for different purposes and calculations in food science and engineering, in managing agrobiodiversity and plant breeding, as well as in food regulatory aspects.

Today, food system sustainability is questioned to better address the SDGs, as are consumers’ dietary shifts driven by environmental concerns [7]. The interconnection between public health and environmental issues is more and more acknowledged and translated into action [8], while FDBs’ gaps have been noticed at the level of the environment-public health nexus [9]. Moreover, the strategic trend of using food by-products as ingredients in other foods (secondary raw materials) seems to be insufficiently addressed by existing FDB. The importance of FDBs is such that inaccurate food composition data can result in incorrect policies (regarding nutritional guidelines and the agri-food system), misleading food labelling, incorrect health claims, and inadequate food choices by the consumers, especially concerning industrially processed foods with added salt, fats, and/or sugars. Therefore, the awareness of relevant new trends and the adjustments to address them is as important as the frequency of FDB’s data update.

A comprehensive review on the production, management, and use of food composition data was released by the FAO (United Nations Food and Agriculture Organization) in 2003 [10], dedicating one chapter to possible limitations of FDB. However, the nexus between food, health, and the environment was not considered because there was little or no awareness yet about it, since the agreement on 2030 agenda only took place in 2015 [11].

According to the FAO [10,12], the three pillars of FDBs should be: (a) the existence of international standards and guidelines for food composition data; (b) national and/or regional programs supporting the regular update of FDB; (c) professional training in aspects related to food composition. In order to ensure these foundations, InFoods (International Network of Food Data Systems) was established in 1984. This FDB is based in regional nodes, under a global coordination, and acts as a network of experts and as a taskforce to respond to users’ needs, database content, organization, and operation, etc. InFoods keep standards in food nomenclature, terminology, and classification systems, in food component identifiers (tag names), in exchange of data between FDB, and in data quality [12]. In addition to its role in setting standards, the FAO/WHO Codex Alimentarius also keeps specific databases, notably on pesticides residues in food and on veterinary drug residues in food [13].

Whilst many countries maintain their own FDB, despite the broad variation of richness and adequacy, the majority of countries keep incomplete, outdated, and/or unreliable food composition datasets or none at all, as further detailed in Section 4.6, dedicated to national FDBs. In such cases, data need to be borrowed from other sources, and the international network of FDBs is, therefore, very important. A list of software tools to assist in nutrient intake estimations and in planning diets is provided in the InFoods webpage, in addition to specific software tools for labelling or for the calculation of food supply/availability [12].

Relevant information on food composition can be retrieved from the FAO [12], EuroFIR [14], USDA [15], and others. It is noteworthy that some national FDBs comply with international standards and are accessible online, in English. That is the case of ANSES-CIQUAL [16] and Frida Food Data [17], whose outstanding dimension, updates, and ease of use turn them into reference databases at the international level. Many other national databases are freely accessible online, in English. Even when their scope is limited, they can be valuable sources of information on specific/ethnical foods, following new trends on diets in compliance with the updated double pyramid model, which relates to the health and environmental impacts of diets [18]. The formats and variability of national FDBs are further discussed below.

The scope of this critical review is to provide new information on the most prominent FDBs freely available online and in English and to discuss their current and future uses, as well as their advantages and limitations in some current applications, e.g., their potential link with human health and their use for preventing chronic diseases.

The current work provides relevant information and links for prominent FDBs and discusses some of their gaps and trends. The need for environmental indicators linked to foods and the coverage of secondary raw materials are argued, and ways on how FDBs can offer better tools for action in the public-health, food, and environment nexus are discussed.

User recommendations and instructions as well as the cybersecurity aspects of FDBs are out of the scope of the current work.

## 2. Main Features and Historical Background

Originally, FDBs existed only in printed form, with the oldest ones dating back to the early 1800s. According to Church [19], the first food composition table dates from 1818, and it was elaborated in the form of a ‘nutrition scale’ aiming at managing food supply in prisons. Early in the 20th century, the USA pioneered standards and regulations aiming at controlling fraud and food safety, and as a result, the USDA’s FDBs are among the most important and comprehensive in the world [15].

The FAO also established an important milestone in this regard when publishing ‘Food Composition Tables for International Use’ back in 1949, to assist in the assessment of food availability at the global level, on a per capita basis, a tool that evolved into today’s food balance sheets, an interactive online tool compiling data on food availability worldwide [20]. The evolution of standards and definitions always have accompanied the pace of growing information, thus scouting and steering its usefulness, a basilar principle, which is more than valid when dealing with Big Data and machine learning algorithms. FDBs continue evolving, as does the knowledge on the chemical nature of food components and the mechanisms by which they exert influence on health and disease. FDBs remain central in nutritional research and guidance, despite the increasing awareness on the complexity and knowledge gaps of the role of food components and their interactions within food matrix [21], suggesting that a nutrient does not have the same health effects depending on the matrix in which it is embedded [22]. Because of that, FDBs are more and more comprehensive and interlinked, providing information on a growing list of features.

Besides whole food composition databases, some specialised ones, generally concerning one class of compounds, are accessible to researchers and other interested parties. In this scope, two classes of compounds have emerged recently: bioactive molecules (such as polyphenols) and microbial metabolites (e.g., butyric acid, accumulated during food fermentations and found to be beneficial in the gut). We open, herein, a parenthesis to categorize both types of compounds, because they have been increasingly noted in innovative foods that highlight health-related aspects.by. In the words of Biesalski et al. [23], a ‘bioactive compound’ is a ‘compound that occurs in nature, part of the food chain, and that can interact with one or more compounds of the living tissue, by showing an effect on human health’. As a consequence, bioactive compounds in a food are chemically defined molecules with a proven function in the body and encompass vitamins, minerals, polyphenols, and others. Bioactive compounds are sometimes named as ‘nutraceuticals’, and there is some confusion around these concepts. According to Heinrich [24], the term nutraceutical is often misused as a synonym of ‘functional food’ and ‘dietary supplement’. Still, according to the same author, ‘functional foods’ are foods that are part of a diet for which scientifically assessed health benefits are acknowledged, sometimes in the form of health claims. That is the case of the so-called ‘function claims’ in Article 13 of Reg. (EC) 1924/2006 and of ‘risk reduction claims’ in Article 14 of the same European regulation [25].

The designation ‘dietary supplements’ corresponds to ingestible preparations (whether synthetic or extracted from natural sources), which are consumed to supplement the diet, with the intention of conveying extra health benefits, or in balancing a (nutritionally poor) diet.

On the other hand, the ‘Nutraceuticals’ designation refers to substances with biological functions that are derived only from foods. Both dietary supplements and nutraceuticals may, thus, refer to products that are consumed in a form that resembles a medicine, and both are sold over-the-counter (OTC). Distinguishing these concepts can be further complicated by the fact that many substances fall within all three categories (functional food, nutraceutical, and dietary supplement). That is the case of beta-carotene, which occurs naturally in fruits, vegetables, and grains, but it can be also synthesised and, thus, also be sold as a dietary supplement and as a nutraceutical. Hence, the commonly found designation of ‘superfoods’ addresses such cases, although it is equally confusing and potentially misleading. Superfoods, functional foods, and nutraceuticals are commonly advertised as having remarkable health claims, such as being able to slow the aging process, having anti-tumoral properties, or in tackling obesity. Such claims are often problematic and difficult to substantiate. From a regulatory point of view, and still according to Heinrich [24], since foods themselves are not considered as therapeutic agents, therefore the claim that nutraceuticals or functional foods can treat disease cannot apply to a food substance.

The second food-related trend, the focus on microbial metabolites, is at an earlier research stage, and despite some penetration in the market (e.g., probiotics), the reach of related (mis)information is currently not significant.

## 3. Current Uses, State-of-the-Art, and Future Challenges of Food Composition Databases

The reference FDBs that were once tables on paper and later on physical digital supports are nowadays easily accessible online, holding and managing large quantities of data and metadata that can be inspected and downloaded. As previous versions, online FDBs mostly detail the composition of fresh produce as well as branded foodstuffs, discriminating energy sources and macronutrients into their components (e.g., amino acids, sugars, starch, fatty acids), as well as minerals (e.g., calcium, iron, sodium) and vitamins. Often, information on other features, as the content of dietary fibre and relevant bioactive constituents (e.g., carotenoids, polyphenols) is also included, and recently, more and more information has been made available, in pace with the development of convenient interfaces to access and use it.

FDBs have been evolving in adapting new ICT tools. A trend in establishing connections between different databases can be observed, thus expanding the available information while allowing the access either by specifically designed algorithms or by individual discrete users making simple searches.

Connections between FDBs complement information about a certain food or about the food sources for a certain compound; for example, bioactive compounds are included in the eBASIS database, in the US isoflavone database and in the French Phenol-Explorer database, all linked to EuroFIR and to FoodData central, as detailed below.

FDBs’ interlinkage adheres to agreed international standards and guidelines, which are of the competence of InFOODs, the International Network of Food Data System from the FAO (UN, Food and Agriculture Organization). It acts as a network of regional datacentres with a central coordination, as well as a forum for the international harmonization and support for food composition activities. InFOODs aims at linking agriculture, biodiversity, food systems, health, and nutrition to achieve better nutrition worldwide. The network regularly issues publications on food composition and other food-related aspects, and its webpage provides access to searchable FDBs [12].

**Figure 1:**
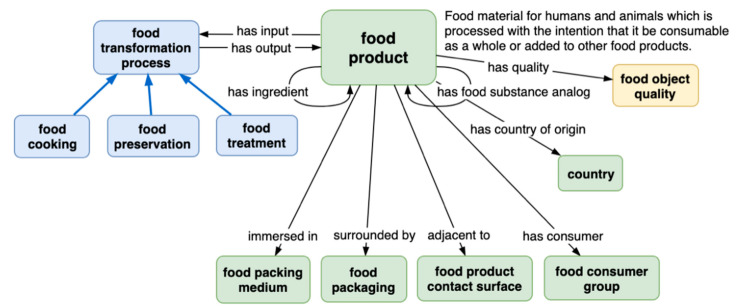
Some facets provided in FoodOn and their relations to a certain food product, of which primary objective is to provide the vocabulary to describe a given food. Reprinted with permission from ref. [26,27]. 2017. Roger A Smith (cc-by-sa/2.0).

The standardization and harmonizing of food composition data from different countries with distinct metadata are essential to ensure efficient data linkage and the retrieval of information. Hence, tools and procedures have been developed aiming to guarantee interoperability between the databases. Langual is such a tool [26]. It is a food description thesaurus that stands for ‘langua alimentaria’ or ‘language of food’ and provides a standardised language for describing foods, specifically in classifying food products for information retrieval. Each of their over 40,000 foods is described by the means of numerical attributes on food composition (nutrients and contaminants), food consumption, and legislation. Langual establishes a correspondence between these food attributes (descriptors) and common language terms in different natural languages [26]. This important tool facilitates the linkage to many different food data banks from different countries, interpreting distinct designations and resolving ambiguities to ensure the correspondence between food and their attributes, thus contributing to coherent data exchange [27]. The food indexing system of Langual already considers food source (e.g., animal or plant species), food preservation (e.g., fresh, frozen), cooking, packaging, etc. However, the next generation of this European FDB thesaurus is even more complex and comprehensive. This global initiative under development—FoodOn—deals with a very comprehensive semantics encompassing descriptors for food safety, food security, agricultural practices, culinary, nutritional and chemical ingredients, and processes [26,27], as can be overviewed in Figure 1.

**Figure 2:**
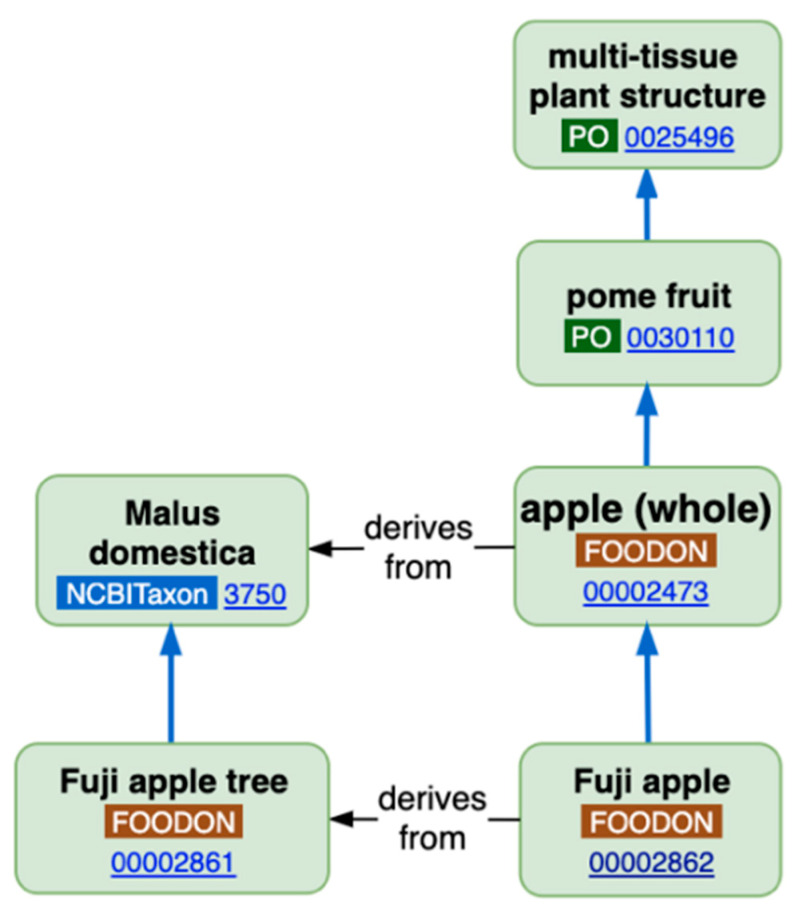
A basic food product, in this case an apple, can be a simple anatomical part, in this case a pome fruit, deriving from a particular plant species (*Malus domestica*) of a specific variety (Fuji). Reprinted with permission from ref. [26,27]. 2017. Roger A Smith (cc-by-sa/2.0).

The detail of such descriptions and relationships can be better understood by observing Figure 2, which refers to an apple. The degree of detail may increase, for example by adding information about ripeness at harvesting. Finally, it is worth mentioning that the Joint Food Ontology Workgroup GitHub (of FoodOn) is working to provide vocabulary for nutritional analysis, such as chemical food components relevant to the diet, as well as many aspects important to research. FoodOn relies on academic curators and some funding agencies’ grants, mostly from Canada [28].

Since 2011, EFSA also maintains FoodEx2, a food classification and description system covering different food safety domains, notably including a description system for exposition assessment. The application range of FoodEx2 encompasses feed additives, food contact materials, food improvement agents, and pesticides [29].

Experimental science advances are based in data, including from FDB, and such figures are commonly fed into models, producing results from which conclusions are withdrawn. Nowadays, these processes can be easily automated by using a bot/API to download data from FDB, which can then be analysed with the assistance of an AI, allowing for instance rapid identification of patterns and trends. With more or less automation, the ability to provide reliable and significant results rely on the research’s rigor and methodologies, as much as on the rigor and detail of the semantics and structure of the database from where the information was withdrawn. Specially developed apps may provide insights on more obvious relationships (e.g., between dietary intakes and health) or less obvious relationships (as between food composition and climate change). So, besides the traditional use in assessing nutrient intakes for diet planning, FDBs can have many more applications for different users in the food value chain, facilitated by IT tools that make it easier to manage and analyse large quantities of data and information. FDBs can, thus, be important tools for exploring the relationship between foods, diets, and nutrients’ intake, regarding nutritional needs and micronutrient deficiencies; yet, a need to better categorise bioactive compounds in foods is emerging, as state-of-the-art knowledge has been disclosing more and more compounds from foods with important physiological roles. Another emerging trend relates to the environmental impact of foods and attempts in systematizing available information are mentioned below (see Section 4.7.3). The key nutritional components found in FDBs are only a few among the more than 26,000 distinct, definable biochemicals present in our food that remain unquantified [30].

Whole food databases are described below and summarised in Table 1. The inclusion criteria were ‘freely accessible online’, in ‘English’, and ‘providing extensive datasets as well as corresponding metadata on food composition’, while exclusion criteria were ‘not in English’ and/or ‘absence of online access and/or information not easily accessible’ and/or ‘pay-per-use/subscription service’ and/or ‘not updated regularly’.

## 4. Main Whole Food Composition Databases

As referred above, the main food composition databases have been enriched with more and more information about food constituents and linkages to different databases. For example, FDBs discriminating nutrients and components of a given food, from fresh product to packed branded foodstuffs (e.g., EuroFIR), are linked to a second type of FDB, which is based on inspecting a wide range of foods for a given nutrient or a certain molecular family of compounds (as is the case of Phenol Explorer). A third type of specific FDB is the object of growing interest—that is, the case of HMDB (see below) exploring the interaction of food components, at the level of gut microbiota, and of metabolites, toxins, and specific compounds (biomarkers) at the cellular, organelle, or pathway level [31,32,33,34].

### 4.1. Food Data Central

The United States Department of Agriculture (USDA) manages and maintains FoodData Central [15], a platform providing access to distinct types of data on nutrients and other food components, including Foundation Foods, National Nutrient Database for Standard Reference (SR Legacy), Food and Nutrient Database for Dietary Studies (FNDDS 2017–2018), and Experimental Foods. The DB platform is noteworthy for providing different types of searches (by component or by food), which may encompass a combination of databases. Metadata are provided, including the number of samples, sampling location, date of collection, analytical approaches used, and if appropriate, agricultural information (e.g., genotype and production practices—intensive, organic, etc.). In respect to Experimental Foods, it is noteworthy that they are meant for research purposes and described foods may not be available in the market. The corresponding database includes data from multiple sources to allow users to examine a range of factors that may affect the nutritional profiles of foods and resulting dietary intakes, as well as the sustainability of agricultural and dietary food systems. This FDB is available at https://agcros-usdaars.opendata.arcgis.com (at the date of the current publication), accessed on 17 August 2021, and the user is able to explore data (referring to US) by topic or by location, for example [15].

### 4.2. CIQUAL—French Food Composition Table

CIQUAL is an open access French FDB [16], covering a wide range of the most consumed foodstuffs in France. This reference database on the nutritional composition of foods is maintained by the Agency for Food, Environmental and Occupational Health and Safety (known by the acronym ANSES). This FDB was updated in 2020 and provides the levels of macro (lipids, fatty acids, carbohydrates) and micronutrients (vitamins, minerals, etc.) of more than 3185 foods and 67 components. The main axes targeted by CIQUAL are the input and management of a reference database relating to the composition of foods, the contribution to the assessment of nutritional risks, and the communication and dissemination of validated data to the greatest number of users (encompassing researchers, nutritionists, food manufacturers, and consumers). In the context of the present work, this database is herein described in more detail, to illustrate the general structure of whole food FDBs, sharing main features and functionalities, essential for interconnections between databases, as explained above.

According to ANSES, finding nutritional information can be carried out by looking for the food in question or by food category. Food categories are classified into eleven food groups:

- Starters and dishes, which in turn divide into six sub-groups: mixed salads (21), soups (46), dishes (159), pizzas, crepe and pies (47), sandwiches (40), savoury pastries, and other starters (24);
- Fruits, vegetables, legumes, and nuts: divided into vegetables (303), potatoes and other tubers (51), legumes (38), fruits (170), and nuts and seeds (52);
- Cereal products: pasta, rice, and grains (71), breads and similar (56), and savoury biscuits (18);
- Meat, egg, and fish: of which the largest sub-groups include cooked meat (133), raw meat (162), delicatessen meat and similar (173), other meat products (16), fish, cooked (63), fish, raw (106), seafood, cooked (24), seafood, raw (25), fish products (56), eggs (24), and meat substitutes (6);
- Milk and milk products are divided into four sub groups;
- Beverages, including water, alcoholic, and non-alcoholic drinks;
- Sugar and confectionery, including products such as jam, sweet biscuits, cakes, and pastry, etc.;
- Ice cream and sorbet, presented as ice cream (11), sorbet (5), and frozen desserts (12);
- Fats and oils (75), such as butters, vegetables oils, margarines, fish oils, and other fats;
- Miscellaneous group exhibit sauces (75), condiments (17), cooking aids (12), salts (6), spices (25), herbs (28), seaweed (17), foods for particular nutritional uses (5), and miscellaneous ingredients for vegetarians (26);
- Finally, the group of baby foods represented by four sub-groups: baby milk and beverages (17), baby dishes (13), baby deserts (5), and baby biscuits and cereals (4).

The nutritional information of each food product is given by a table either in detailed composition or in basic composition. In the case of detailed composition, the estimated energy provided from fibres is also included (based in Jones’ factor). All the nutrients likely to be present in the food are provided by the table and are expressed in g/100 g or g/100 mL of the edible part. Lipids are detailed by the fatty acid profile (saturated and polyunsaturated). Fibres, water, starch, vitamins, and oligo-elements are all exposed, but not for all foods systematically, and the level of detail may vary. The data source of each compound is also mentioned by CIQUAL, and it may come from different sources, given the interlinkage between FDBs. Thus, data are a compilation between a sampling plan and analyses launched each year by ANSES on 60 to 80 foods in collaboration with subcontracted laboratories, plus data from OQALI (a French project, which aims at monitoring changes in processed foods supply available on the French market), research programs carried out jointly with external partners, information from scientific literature and laboratory, and finally, data from foreign food composition tables [16].

### 4.3. EuroFIR, European Food Information Resource

EuroFIR, European Food Information Resource [14], is an independent food composition resource in Europe bringing together food composition datasets from 26 European Countries, Canada, the US, New Zealand, and Japan. It is currently a non-profit international organization that resulted from a network project, Network of Excellence (NoE) comprising of 48 partners from academia, research organizations, and small- and medium-sized enterprises (SMEs). EuroFIR is a food composition table or database providing detailed information on the nutritional composition of foods, typically energy, macronutrients (e.g., protein, carbohydrate, fat) and their components (e.g., sugars, starch, fatty acids), minerals (e.g., calcium, iron, sodium), and vitamins [14]. One of its tools, Food Explorer, is an interface that allows to simultaneously search information about food composition data from most of the available databases from the EU, Canada, USA, New Zealand, and Japan. Food Explorer allows searches by food names or by nutritional groups with the unique ability to allow comparisons of attributes’ values of foods from different countries. Another relevant tool in this FDB is Bioactive Substances in Food Information Systems (eBASIS), which is a compilation of food composition and their biological effects. Such data are extracted from peer-reviewed literature as raw data and critically evaluated, thus relying on the curation work of experts.

### 4.4. FoodDB

The Canadian database, FoodDB Version 1.0, 2021, is licensed under a Creative Commons Attribution-Non-Commercial 4.0 International License, and it is supported by the Canadian Institutes of Health Research, Canada Foundation for Innovation, and by The Metabolomics Innovation Centre (TMIC) [35].

This FDB supplies extensive data on food constituents, chemistry, and biology, providing information on both macronutrients and micronutrients, including many of the constituents that give foods their flavour, colour, taste, texture, and aroma with detailed compositional, biochemical, and physiological information (obtained from the literature). Searches can be made by food source, name, function, or concentrations, and the FDB content can be accessed from the Food Browse (listing foods by their chemical composition) or from the Compound Browse (listing chemicals by their food sources), according to the user’s preferences. A section called ‘reports’ is noteworthy, since it concerns monographies of a list of foods featuring composition and nutritional and health benefits, based on scientific literature review [35].

### 4.5. Frida Food Data

The database Frida Food Data (frida.fooddata.dk), also known as DTU foods [17], is managed by the National Food Institute with the Technical University of Denmark (DTU) allowing public access to information about foods available in Denmark. The FDB also relies on the cooperation of stakeholders as food industries and retailers, as well as scholars and the Danish Veterinary and Food Administration. Metadata (as the number of samples and their source) are included in registries encompassing more than 1000 food items.

The information above is summarised in Table 1 presenting some features of the most utilized food composition databases, for whole foods, easily and freely accessible online, in English.

The FDBs listed in Table 1 follow international standards and are interconnected thus providing access to reliable, comprehensive information on foods serving most common purposes.

In view of the current transformation of food systems in meeting the 2030 agenda, average global data on food composition may not be enough, as consumers are being encouraged to prefer healthier foods respectful of their food cultures and the environment [18]. Such changes will sooner or later reflect the level of the usage of FDBs, and consequently, the inspection of food habits linked to traditional balanced diets may direct the spotlights towards certain FDBs of national ambit. The panorama is currently not so encouraging because of the great variation observed from country to country, as illustrated in the section below.

### 4.6. National Whole Food Composition Databases

National FDBs, where they exist, vary widely in the extent of provided information, standardisation at various levels (see Figure 1; Figure 2), and the ease of access (including the language). Thus, starting by the British food composition table, obviously in English, in the United Kingdom, Public Health England (PHE) is responsible for maintaining food composition data relating to nutrients (macronutrients, e.g., fats, protein, carbohydrates as well as their micronutrient content, which includes vitamins and minerals) mostly from analysing foods commonly consumed in the country. The results are published as McCance and Widdowson’s ‘The Composition of Foods’—the UK food composition tables. The Composition of Foods Integrated Dataset (CoFIDS) is a nutrient dataset for 2898 foods and 303 others in the ‘old foods’ file, comprising 185 individual nutrients. CoFIDS is searchable online and can be downloaded free of charge in MS Excel or Ascii format, and it was first published in 2008 (https://fdnc.quadram.ac.uk/ accessed on 17 August 2021), available online at the date of this publication.

PortFIR is the Portuguese national food composition database for the most consumed foods in Portugal. The data cover about 42 nutrients ex. energy, macronutrients, fatty acids, vitamins, and minerals (http://portfir.insa.pt/ accessed on 17 August 2021), available online at the date of this publication. The information is classified into groups and sub-groups according to the FoodEx2 classification and description system (http://www.efsa.europa.eu/en/datex/datexfoodclass accessed on 17 August 2021), available online at the date of this publication from the EFSA. The PortFIR FDB is free online, displayed in English, and allows searches as well as downloading in Excel format [36].

Similarly, the Turkish food composition database, an open access digital platform, ‘Türkomp’ (http://www.turkomp.gov.tr/main accessed on 17 August 2021), available online at the date of this publication provides a considerable dataset and information related to the nutrients, composition, and energy values of processed or unprocessed agricultural products that are produced and consumed in Turkey. Türkomp exhibits 63,000 data entries on the nutritional and energy value of 100 food components belonging to 580 foods from 14 food groups [37].

As referred above, it is rare to find suitable food composition tables of reliable and updated contents from developing countries, and to illustrate such situations, a few examples are herein presented.

Thus, in Morocco, a country integrating the UNESCO’s list of countries that safeguard the Mediterranean diet as intangible heritage of humankind [38], the development of a national composition table dates back to 1977 by the Ministry of Agriculture of Morocco and was revised in 1984 by El Khayate [39]. Since then, no updates have been made. Recently, a multidisciplinary team of Moroccan and international experts worked on updating the food composition table, in order to supplemented it with high quality composition data. The consolidated version includes information on 38 nutrients, from 587 food products commonly consumed in Morocco. This update represents a 79% addition of foods, and according to the authors, 7% of nutritional values come from Moroccan data sources and 93% from international data sources, mainly from Tunisia, West Africa, France, the United Kingdom, and the United States [40]. The updated version provides information on foods and dishes commonly consumed in Morocco and can be used as a tool to promote nutritional research and to design public health strategies.

Another common situation with national databases of developing countries can be illustrated by the Tunisian food composition table, which displays the 240 foods and dishes usually consumed by Tunisians. The table corresponds to 95% of the food needs of the entire Tunisian population. It includes, for each food, the energy value as well as the content in 34 nutrients, expressed per 100 g of the raw edible part. This table is presented in the form of a book produced by a group of nutritionists from the National Institute of Nutrition and Food Technology (INNTA) who were supported by French and Belgian experts within the framework of the European project ‘Impact of transitions epidemiological studies on health in North African countries’ [41]. Another common situation corresponds to the composition table of foods from the Republic of Bahrain, which is a printed book not so regularly updated and hardly available. This database brings together 150 raw and ready-to-eat foods and composite dishes according to standardized methods. This list includes cereals and grain products, bread and bread products, fruits, vegetables, legumes, nuts and seeds, meat, poultry and eggs, fish, milk and dairy products, fats and oils, herbs and spices, beverages, local and western fast foods, etc. The table provides data for proximate composition, three minerals (calcium, phosphorus, and iron), and five vitamins (retinol, thiamine, riboflavin, niacin and vitamin C) expressed per 100 g of edible portion [42].

Similarly, the Chinese food composition database is given by a printed book, not necessarily in English [43].

As the reader can easily deduce, the randomness of updates, the limited access, and the absence of English versions can be strong limitations to the use of national FDBs in disclosing specific food habits and/or the composition of particular food items.

In addition to free access institutional databases, a growing number of commercial customized applications have been appearing in the market. Such apps or so-called food databases mainly encompass different types of software to assist food formulation and labelling, dietary features, and recipe analysis, as well as fitness apps. The access is reserved and includes consultancy support services.

An example of a privately owned FDB, with an associated API, is offered by Edamam, a company that provides access to a food and grocery database with close to 900,000 basic foods, restaurant items, and consumer packaged foods available on the website, at the date of this publication, https://developer.edamam.com/food-database-api (accessed on 17 August 2021). The Food API provides a filter to sort data by diet and health, determining dietary, allergy, and nutrition labelling, based on the food’s ingredients. Over 70+ claims are automatically generated such as peanut free, shellfish free, gluten free, vegan, and vegetarian.

Edamam also provide data for basic foods (as flour and eggs) for calories, fats, carbohydrates, protein, cholesterol, sodium, etc., for a total of 28 nutrients.

### 4.7. Specific Purpose’s Food Databases

#### 4.7.1. FDBs Directly Related with Human Metabolism

The food we ingest is expected to interact at the level of the gut microbiota, and thus, considering the scenario of metabolic pathways and the benefits of bioactive compounds in humans, Durazzo et al. [44] noted the database Human Metabolome Database (HMDB) version 4.0, also originating from Canada, and supported by the same organizations as FoodDB vs.1. This database, HMDB, contains detailed information about small molecule metabolites found in the human body aiming to be an input for studies in metabolomics, clinical chemistry, biomarker discovery, etc. This database encompasses data of different kinds: chemical, clinical, and molecular biology/biochemistry data, notably more than 100,000 metabolite entries (water-soluble and non-polar metabolites) either abundant (>1 μM) or rare (<1 nM), which are linked to almost 6000 protein sequences. Even if this database does not directly reflect food composition, it is of undoubted interest in nutritional studies to assess how a food or a diet might influence metabolism, either in a positive or unhealthy way. The HMDB supports text, sequence, chemical structure, and relational query searches, and it is linked to other databases whether on drugs, toxins, pollutants, or on nutrients and food additives [31,32,33,34]. At https://hmdb.ca accessed on 17 August 2021, available online at the date of this publication it is possible to browse metabolites, pathways, etc., as well as performing advanced searches based on molecular mass, chemical structure, or text queries.

Another freely accessible data resource of the same kind is MGnify, an EMBL-EBI online resource containing Human Gastrointestinal Protein catalogue and a dataset on the Human Gastrointestinal Genome, allowing researchers to compare their findings on microbial genomics and proteomics with existing datasets. MGnify has been growing, and promoters would like to close knowledge gaps, such as the variation in bacterial diversity across different human populations [45].

The Sydney University Glycaemic Index Research Service (SUGiRS) produced a free database that gives the glycaemic index of any food inserted on their search engine available, at the date of this publication, on their website https://www.glycemicindex.com (accessed on 17 August 2021) and the Gluten-Free Food Database (Austria) provides quantitative information of macro- and micronutrients of the gluten-free products. This database can be accessed via the science collaboration platform, Open Science Framework, upon registration, and it also accepts contributions to the dataset [46].

#### 4.7.2. FDBs Concerning Food Processing

In order to process safe food, several hours of research are needed when searching for the precise thermal processing parameters; D-value and z-value parameters that describe the characteristics of thermal death of food target microorganisms, for the ingredients or final food products, are not always easily found. The Lemgo D- and z-value Database for food, a project of the Institute for Food Technology NRW (ILT.NRW) at the OWL University of Applied Sciences and Arts, supplies information on these parameters, to design pasteurization or sterilization processes with a main focus on beverage spoiling microorganisms. Additional information is given on parameters known to have an effect on the D- and z-values like pH, Brix and a_w_ value. The data are sorted by the species of microorganism and their medium, and on the experiments from which these data originated or a cluster of relevant data [47].

Another very important database for food engineers is the Database of Physical Properties of Food is available online, at the date of the current publication http://www.nelfood.com, (accessed on 17 August 2021); nelfood.com grew out of the Physical Properties of Food Data Base project that started to collect and publish on the internet reliable and useful data on Physical Properties of Foods. This project was managed by Dr Paul Nesvadba with internet work done by NEL, and it was partly funded by the EU and partly sponsored by companies such as Nestle, RHM, and Unilever. It is only available to subscribed members that may search 11,094 bibliographic references, 1519 materials, and 1694 experiment datasets. These datasets range over 24 food categories encompassing 249 food subcategories and 260 physical properties. NELFOOD Database covers five main groups of physical properties: (1) Mechanical and Rheological Properties of Foods; (2) Sorption and Mass Diffusion Properties of Foods; (3) Electrical and Dielectric Properties of Foods, and (4) Optical Properties of Foods [48].

#### 4.7.3. FDBs Concerning Environmental Impact of Foods

Generally speaking, current food systems are operating out of planetary boundaries, with agriculture being a top driver for biodiversity loss, using water above the natural capacity of replenishment, causing soil degradation, pollution, and more [49,50]. The urge of the food systems’ transformation is such that, among many initiatives, the UN organised a food system summit in 2021 (https://www.un.org/en/food-systems-summit accessed on 17 August 2021), available online at the data of this publication and the European Union issued a climate law that binds the EU Institutions and the Member States to take the necessary measures to reach carbon neutrality by 2050. On the other hand, a growing awareness from consumers about the impact of their individual food choices in their health and the environment has been registered [51]. Shifts in food habits may fuel the desired changes, but the commitment of food producers is key. Business pledges need to be underpinned in well-established targets and robust metrics fed with comprehensive information on the food–environment nexus. Despite the still existing gaps, efforts in compiling information are many, and advancements of FDBs in integrating data on the environmental footprint of foods are to be expected.

In respect to the 2030 agenda, the Sustainable Development Report, by Sachs et al., [52] provides interactive dashboards with visual representation of performances by SDGs to identify priorities for action. One of such priorities is tackling food loss and waste for which the FAO maintains a database in connection to tools to track progress, available online at the date of this publication, http://www.fao.org/platform-food-loss-waste/flw-data/en/ (accessed on 17 August 2021), at the date of this publication. The food loss and waste database contains data and information from various sources, measuring food loss and waste across food products, stages of the value chain, and geographical areas, also presenting underlying causes, according to the literature [53].

Only a few FDBs present datasets on the environmental footprint of foods or are useful for its assessment. One of them is ‘Experimental Foods’ from USDA (see Section 4.1) that contains information on environmental inputs and outputs on the supply chains, etc.; however, it is not necessarily publicly available [15]. A dataset on food environmental impacts through producers and consumers was published by Poore and Nemecek in 2018 [54]. The ADEME (the French Agency for Ecological Transition) recently launched Agribalyse, a food database providing an environmental score (Ecoscore) for 2500 food products based on their life cycle analysis (LCA). However, this database has already been criticized, notably by institutions promoting organic agriculture, for favouring intensive farming systems and not taking into account the consequences on biodiversity, animal well-being, or the impact of pesticides [55]. More generally, LCA, on which Agribalyse is primarily based, has already been questioned for being unsuitable for comparing farming systems. Thus, an improvement of such a tool would be necessary to inform public policies [56].

## 5. Main Limitations of Food Databases: Missing Dimensions for Human Health?

First, beyond only nutrients, foods are interlinked with cultural identity while playing a key role in many local economies, as highlighted by Dembska et al. [18] in their double pyramids models connecting food culture, health, and climate. These authors and others [18,57,58,59,60,61] call attention to the need of leveraging the various dimensions of foods, which are closely related, under the so-called one-health approach [18,57].

If FDBs are specifically useful for balancing a diet for nutrient composition and fully addressing nutritional needs in human studies, they, however, reflect a reductionist view of foods, viewed as only the sum of nutrients [61], not considering the food matrix effect, and hence, the degree of processing [22]. Therefore, to be a relevant tool regarding human health in the long term, their data should not be used alone, but other parameters should be also considered, such as food form and degree of processing, together with other important food properties.

For example, the newly developed Siga score [59] is hierarchically combined with the first degree of processing, then the food matrix effect, added salt, fat, and/or sugar, and the number of markers of ultra-processing (including some cosmetic additives and non-additive markers) [60]. To be elaborated, this score typically needs not only the food composition data, but also the list of ingredients and the presence or not of added sugar, salt, and/or fat. Such a hierarchical and holistic score should be more considered, because, in the end, it is related to global (environmental and human) health [18,57,60,61]; whereas, food composition only is insufficient to address diets from the global or one-health perspective as needed (e.g., compliance with European Climate Law).

### 5.1. The Matrix Effect Is Not Considered

First, the whole food potential is not only reflected by its nutrient composition. Whole foods are first complex matrices, which govern the health effects of nutrients [22]. Besides, food form matters for human health, be it solid, semi-solid, or liquid. It should be emphasized that interactions between nutrients within the food matrix participate in a food’s health potential, including notable food chewing and satiety [62], nutrient kinetics of release, and final bioavailability. For example, the calcium of dairy products is only 20–40% bioavailable; therefore, 120 mg of calcium in a yogurt corresponds to around 36 mg being bioavailable, with the remaining fraction reaching the colon [63]. The same is true for the lipid content of a whole almond, which is not fully available [64]. Otherwise, within an extruded-cooked breakfast cereal, wheat flour, and/or maize semolina behave close to simple sugars in human organism with a glycaemic index above 80 [65], and so on for most of nutrients, depending on the food form and on the impact of processing on the food matrix. Such fundamental physiological properties go beyond the simple nutritional composition, which leads to the hypothesis that chronic diseases have more to do with highly degraded and artificialized food matrices than with the food composition itself [22].

### 5.2. Some Important Bioactive Compounds and Food Properties Are Still Missing

Another limitation is often observed worldwide and consists of missing values for some important key nutrients, e.g., lipotropic compounds (such as choline, betaine, and myo-inositol) and phytic acid, but also for other characteristics of nutrients or foods, such as soluble and insoluble fibre (with different physiological effects), resistant starch, and glycaemic index [65]. It is true that some FDBs report choline content such as the USDA Database for the Choline Content of Common Foods, Release 2 (2008) [66], or the phytic acid content such as the FAO/INFOODS/IZiNCG, Global Food Composition Database for Phytate (2018) [67], or the glycaemic index [68], but this should be completed and extrapolated to other FDBs more broadly in the future, e.g., the French CIQUAL database [16].

### 5.3. The Important Dimension of the Degree of Food Processing

Therefore, FDBs must not be considered as a sufficient tool for reaching human health on a long term. Notably, one can fully address one’s nutritional needs and become chronically ill, as is frequently observed in Western countries. This is notably due to the matrix quality of consumed calories, not only the quantity, and this quality may depend on the degree of food processing.

It is noteworthy that food engineers and food technologists have been, in the last three decades, dedicating a great part of their research to studies on reducing the processing load that can be achieved on the application of milder preservation technologies (still called emerging technologies). Such milder preservation technologies can be used alone or combined with less severe thermal treatments, such as high hydrostatic pressure [69], pulses of electric field [70], UV-c radiation [71], thermosonication [72], and others. The optimization of these processes aims at maximizing the retention of food nutrients such as vitamins [73], proteins, and sensory parameters such as texture, colour, and taste, while keeping the product safe [74,75].

On the other hand, in the circular economy model, food industries are expected to play a key role in tackling food loss and waste, which poses the double burden of depleting natural resources and wasting extra energy from production to disposal. Innovations that consist of using by-products of an industry as raw materials of another, as well as recovering nutrients that would otherwise be wasted are emerging tendencies within a biorefinery approach. An illustrative example is reported by Lucarni et al. 2020 [76], exposing a new class of ingredients that may not yet be adequately covered by FDB.

Considering industrially processed foods that are becoming dominant in our diets, in the future, FDBs should also distinguish between ‘natural’ and ‘added’ nutrients whatever they are and indicate the list of additives, as with the Open Food Facts database available online for industrial foods [77] or private pay-per-use food databases that also gives the list of ingredients, e.g., Alkemics and Num-Alim. More specifically, the Open Food Facts database is a collaborative database of food products and is licenced under the Open Database Licence (ODBL). For such foods, the list of ingredients tells us more about their whole nutritional quality (including environmental aspects) than the only composition.

Indeed, it should be underlined that no food is nutritionally balanced (except maternal milk for the growth of the infant), hence the recommendation to ‘eat varied’ at the level of the diet.

## 6. Emerging Applications and Trends of Food Databases

In view of the ongoing changes in food systems, needs for curated and organized information on the composition of food secondary raw-materials, novel foods, and/or sources for nutrients (as insects and microalgae) are expected to be met by FDBs. These challenges may exacerbate existing issues with food data composition. Thus, in addition to the intrinsic features of foods, parameters related to the extraction and analytical procedures should be considered, according to Durazzo et al., [44], as different extraction procedures and analytical techniques and methodologies may lead to different datasets. Moreover, still according to these authors, only a few compounds within a class are investigated, and there are knowledge gaps on appropriate analytical methods for food analysis. The acknowledged complexity of foods (in their multiple dimensions) calls for information on multiple relationships, as the nexus between public health and the environment, or consumer preference and health [51,52,78,79]. Ocké et al. [9], besides identifying some gaps herein mentioned, also refer to the need for FDBs’ adaptation to the rapidly changing food landscape and the need for their improvement and harmonization to enable comparisons of research outputs at international level. More generally, in the near future, there is, therefore, an important need for more comprehensive and holistic FDB, not only addressing nutritional composition, but also other food properties. In this way, FDBs will, thus, constitute more robust tools for tackling global health, but this means a huge scientific work to gather all data, notably when thousands of new industrially processed foods are marketed each year worldwide.

## 7. Conclusions

**Figure 3:**
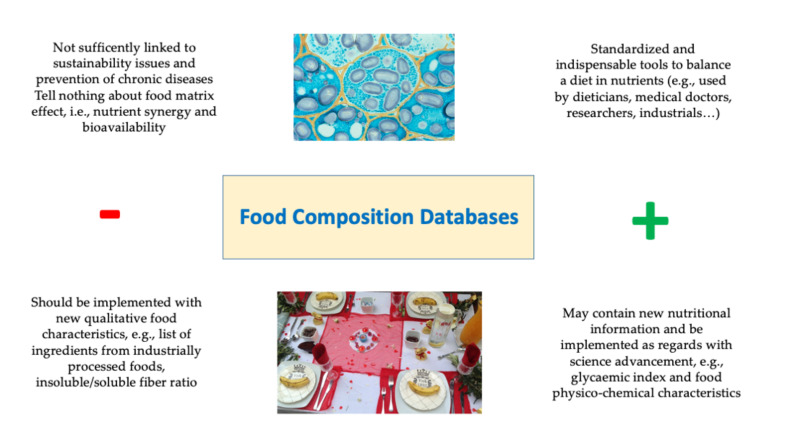
Main features, common uses, identified gaps, and expected trends of food composition databases (original figure by Anthony Fardet; photos: INRAE for bean food matrix under optical microscopy and Amélia Delgado for the meal table).

Food composition data are fundamental information resources to many fields of work, as in formulating and labelling foods, as well as in public health and nutrition. Thus, food industrials, legislators, and consumers all need and/or use reliable data on food composition, provided from FDBs (Figure 3). In other words, nutritional and physico-chemical features of foods are valuable tools for medical doctors and dieticians in prescribing nutritionally balanced and/or low-GI diets, as well as for researchers and industry workers, notably in developing the most nutrient-dense foods.

However, FDBs do have limitations, encompassing variability in the composition of foods between countries, from season to season; food composition depends on the cultivar or variety; manufactured foods of the same recipe may vary from brand to brand and between lots; missing values for some important food characteristics (e.g., list of ingredients for industrially processed foods), etc. In addition, FDBs can only provide an incomplete coverage of foods and/or their nutrients leading to gaps in values, as missing information on some minority compounds (from aromas to chemical contaminants). Despite efforts on updates, data ageing is inevitable due to limited resources.

Food databases have been following the advancements of science, as highlighted above (see Section 3 and Section 4.7), and today’s challenges include adding comprehensive information about the environmental impact of foods, health/sustainability linkages, as well as qualitative features, because food goes far beyond its composition (Figure 3).

Concerning the relevance of FDBs for human health, they only indirectly address a reductionist view of it and should not be used for other purposes than building a balanced diet to fully address nutritional needs and avoid nutritional deficiencies. However, other criteria should also be considered. Most importantly, food composition does not say anything about the nutrient kinetics of release and final bioavailability within the human organism and on health effects in the longer term. Otherwise, due to the increasing marketing of industrially processed foodstuffs worldwide, comprehensive FDBs should probably integrate more of these foods in a near future, together with their corresponding content in additives, aromas, and added fat, sugar, protein, fibre, and salt, to distinguish between the ‘natural’ and ‘artificial’ origins. In addition, other food health potential metrics or indicators such as the soluble/insoluble fibre ratio and/or glycaemic index would deserve to be added in FDBs whenever possible. This could be important issues for the future of this nutritional tool, and this will strengthen their link with human health.

In the end, if nutrient composition is a relevant tool for addressing nutrient needs, it is not sufficiently linked to global health and food system sustainability, and apart for organic plant/animal and some traditional foods that may contain higher nutritional densities (e.g., omega 3 fatty acids and antioxidants), the stronger connexion is between plant versus animal-based foods and with degree of food processing, i.e., at the level of complex foods, a higher scale of observation than nutrients, i.e., more in connection with reality.

## Figures and Tables

**Table 1:** Food composition databases publicly available, in English, allowing data searches and/or download.

Organization	Name of FDB	URL (Available at the Bate of the Current Publication)	Discrimination of Food Composition	Source of Data	Ease of Access	Regularity of Updates	Citation/Site
**USDA**	FoodData Central	https://fdc.nal.usda.gov accessed on 17 August 2021;	Target important components that make sense in each food; highly discriminated	Laboratory analysis by state-of-the-art methods	Search by food name or by component + API for access with proprietary app; instructions and tips provided	Regularly updated (date is shown)	U.S. Department of Agriculture (USDA), Agricultural Research Service. FoodData Central: Version October 2020.:
**TMIC**	FoodB	www.foodb.ca accessed on 17 August 2021	Content range and average values for an extensive list of compounds	Literature and other FDB	Search by food name or browse foods by constituents	Frequency of updates not mentioned (last update in 2021)	www.foodb.ca accessed on 17 August 2021
**DTU food (National food Institute (Denmark)**	Fødevaredata (Frida Food Data)	http://frida.fooddata.dk/ accessed on 17 August 2021	DTU foods’ database—Frida Food Data reflects the food supply in Denmark and targets professionals in food and nutrition	Laboratory analysis	Easily searched by food item (alphabetic order), food group or by parameters, which include waste and added sugar	Updated every few years (last update 29/10/19) and food composition referred to be quite stable over the past 50 years	Food data (frida.fooddata.dk accessed on 17 August 2021), version 4, 2019, National Food Institute, Technical University of Denmark
**EuroFIR AISBL, International non-profit association**	EuroFIR	https://www.eurofir.org/food-information/ accessed on 17 August 2021	The dataset presents energy, macronutrients, vitamins, and minerals as well as other bioactive compounds and daily recommended intakes for selected nutrients	Estimations from FDB by expert panels and targets food and nutrition professionals	Search by food name and by component	Updated regularly (each few years)—last update 21 January	European Food Safety Authority (2013) ‘Food composition database for nutrient intake: selected vitamins and minerals in selected European countries’. Zenodo. doi: 10.5281/ZENODO.438313.
**FAO**	InFoods	http://www.fao.org/infoods/infoods/en/ accessed on 17 August 2021	InFoods is a network bringing together food composition compilers, data generators (e.g., chemists), and data users (e.g., nutritionists, food scientists), and decision makers	food composition database compilers retrieve analytical data on food composition for commonly consumed foods and complemented with other published sources	Datasets are downloadable in xls and pdf formats, as well as searchable with software tools for e.g., dietary assessment, labeling and food supply/availability data	Updated regularly	FAO. 2020. International Network of Food Data Systems (InFoods)
**CIQUAL-ANSES**	French Food Composition Database	https://ciqual.anses.fr/ accessed on 17 August 2021	Average nutritional composition of food consumed in France. Average value of each component, a minimum and a maximum, together with a confidence code (A = very reliable, D = less reliable). Information on a specific component (ex. list of food rich in calcium or poor in sodium)	Compilation of different sources: yearly sampling of around 60 to 80 foods in collaboration with subcontractor laboratory; data from OQALI; research programmes on food composition with external partners. Scientific literature and laboratory reports; foreign food composition tables	Easily searched by food item, food group, or by components	Released every 2 to 4 years	French Agency for Food, Environmental and Occupational Health and Safety. ANSES-CIQUAL French food composition table version 2020.
